# Supplementary material for: Modelling Skylarks (Alauda arvensis) to Predict Impacts of Changes in Land Management and Policy: Development and Testing of an Agent-Based Model
Source: PLoS One. 2013 Jun 6;8(6):e65803. doi: 10.1371/journal.pone.0065803 (PMC3675089; doi:10.1371/journal.pone.0065803)
Supplement: Supporting Information S4 — The skylark ODdox as a zipped archive. (ZIP) [file pone.0065803.s004.zip › Skylark_ODdox/dir_391a3558160bdb967d9c0f4086e982f4.html]

ALMaSS Skylark ODdox: C:/CJT/ Directory Reference


ALMaSS Skylark ODdox
 2.0


- Main Page
- Related Pages
- Classes
- Files

CJT Directory Reference

|  |  |
| --- | --- |
| Directories | |
| directory | MSVC |


- CJT
- Generated on Thu Jan 10 2013 13:15:38 for ALMaSS Skylark ODdox by
   1.8.1.1
